# Nutritional status among primary school children in rural Sri Lanka; a public health challenge for a country with high child health standards

**DOI:** 10.1186/s12889-016-4001-1

**Published:** 2017-01-10

**Authors:** N. P. G. C. R. Naotunna, M. Dayarathna, H. Maheshi, G. S. Amarasinghe, V. S. Kithmini, M. Rathnayaka, L. Premachandra, N. Premarathna, P. C. Rajasinghe, G. Wijewardana, T. C. Agampodi, S. B. Agampodi

**Affiliations:** 1 Maternal and Child Health Research Unit, Department of Community Medicine, Faculty of Medicine and Allied Sciences, Rajarata University of Sri Lanka, Saliyapura, Sri Lanka; 2 Faculty of Engineering, University of Melbourne, Parkville, VIC Australia

**Keywords:** Nutritional Status, Primary school children, Sri Lanka, BMI, Height for age, Weight for age, Thinness, Obesity

## Abstract

**Background:**

Nutritional status of pre adolescent children is not widely studied in Sri Lanka. The purpose of this study was to determine the nutritional status among pre-adolescent school children in a rural province of Sri Lanka.

**Methods:**

A school based cross sectional study was carried out in North Central Province in 100 rural schools, selected using multi stage cluster sampling with probability proportionate to size. Children in grade one to five were enrolled with a maximum cluster size of fifty. Anthropometric measurements were done by trained data collectors and venesection was done at site by trained nurses. WHO AnthoPlus was used to calculate the BMI, height for age and weight for age Z scores. Survey design adjusted prevalence estimates with linearized standard errors were generated using svy function of STATA. Mean haemoglobin concentration (Hb) was calculated using methaeamoglobin method. Screening for iron deficiency and thalassemia trait was done using peripheral blood films.

**Results:**

Height and weight measurements were done for 4469 of children and the Hb data was available for 4398 children. Based on the survey design adjusted estimates, prevalence of severe thinness, thinness, overweight and obesity in this population was 8.60% (SE 0.94), 2.91%(SE 0.74), 2.95%(0.26) and 2.43%(SE 0.92) respectively. Similarly, survey design adjusted prevalence of underweight and stunting were, 25.93% (95% CI 24.07–27.89%) and 43.92%(95% CI 40.55–47.56%). Adjusted mean estimates for hemoglobin was 12.20 (95% CI 12.16–12.24) g/dL. Prevalence of anemia was 17.3% (*n* = 749). Prevalence of mild and moderate anemia was 9.4 and 7.6% respectively.

**Conclusion:**

This study confirms that malnutrition is still a major problem in North Central Province, Sri Lanka.

## Background

Nutritional problems are estimated to be associated with more than one third of global child deaths [1]. WHO, UNICEF, World Bank joint estimates show that globally 161 million children were stunted, 51 million children were wasted and 99 million children were underweight in 2014. In addition, 41 million children under 5 year old were estimated to be overweight [2]. Regional, and country level disparities of nutritional status are high and Asia is having the highest burden of childhood malnutrition.

Childhood malnutrition is significantly associated with adverse health effects during the childhood and reported to be underlying half of the in-hospital mortalities and morbidity due to severe disease including severe malaria, gastroenteritis, lower respiratory tract infections, HIV, and invasive bacterial diseases in children under 5 years [3]. A growing body of evidence suggests that childhood malnutrition leads to adverse health effects during adulthood. A recent study has detected reduced beta cell function in stunted children predicting risk for type 2 diabetes mellitus [4]. Stunted girls were found to have a lower resting metabolic rate and higher rate of weight gain which can result in obesity in later life [5]. Increased systolic and diastolic blood pressure has been observed in malnourished children and in those who recovered from malnutrition after an average period of 6 years. This may represent a risk factor for increased BP later in their life [6].

Latest estimates on child stunting at country level shows that Sri Lanka is doing much better than all the other south Asian region countries [7]. This is on par with other public health indicators such as maternal mortality ratio (32.5 per 100,000 live births) and infant mortality rate (8.8 per 1000 live births). Though the comparative data at national level are satisfactory, country specific data during last two decades clearly show that Sri Lanka is having a very low progress in relation to nutritional indicators since 2000 [8]. Further, very high level of regional disparities is observed within the country due to various socio-demographic and behavioral practices.

Among children under 5 years, number of studies have been carried out during recent years in Southern province [9–11], North Western province [12], among institutionalize children [13] and national level studies [14] including the demographic and health survey [15] showing regional disparities of nutritional status. Nutritional status of adolescent age group is also widely studied in Sri Lanka [10, 16–21] due to unique problems related to this category. However, nutritional problems of pre-adolescent children are not studied widely. Sri Lanka and many other countries do not have national reference data for this age group on growth and nutritional status. Identifying that the focus on some of the age categories is lacking, WHO has included growth standards up to the age of 19 years in revised WHO AnthroPlus, a software for the global application of the WHO Reference 2007 for 5–19 years to monitor the growth of school-age children and adolescents. Purpose of the present study was to describe the growth and nutritional status of pre-adolescent children aging 5–10 years residing in a rural province of Sri Lanka using BMI for age (thinness), height for age (stunting) and weight for height (wasting) using WHO child growth reference data and to evaluate the hemoglobin levels and iron status.

## Methods

### Study design

We carried out a school based cross sectional descriptive study to determine the nutritional status of preadolescent school children in North Central Province (NCP).

### Study Setting

This school based cross sectional, descriptive study was carried out in NCP, Sri Lanka. NCP is divided into 8 administrative zones, which are geographically defined. Altogether, 802 schools are located in NCP with a total number of 251,748 students. Of these schools, 701 are classified as very difficult or difficult schools, based on resource availability, access and number of students in the school. These 701 schools are located in rural and remote areas of NCP with a total student population of 93,243.

### Study population

School children studying in grade 1 to 5 (aging 5 to 10 years) and attending very difficult and difficult schools in North Central Province were the study population for the present study. In Sri Lanka, net school enrolment is rural areas is 99.8 and almost 100% retain in schools at grade five [22]. Thus, a school based study among primary school children was considered as an appropriate design to describe nutritional status of children in the particular age group.

### Study Sample and sampling technique

Our objective was to estimate the age and sex disaggregated data for nutritional status. The number of age categories expected was six (61–131 months with age categories for each 12 months) and the total number of strata within the sample was 12. Of the four main outcome measures of interest (BMI for age, height for age, weight for height and anemia), the lowest estimated prevalence from other studies was for anemia (15%) and we used this number for sample size calculation. With 95% confidence limit, expected prevalence of 15% and a precision of .05, the required sample size was 196. With an estimated design effect of 2 for cluster sampling, 384 students from each stratum were required and the minimum sample required for the study was 4608. With a median of 50 children within the study age range in a given school, and with a margin for none-respondent, the number of schools to be selected was decided as 100.

**Fig. 1:**
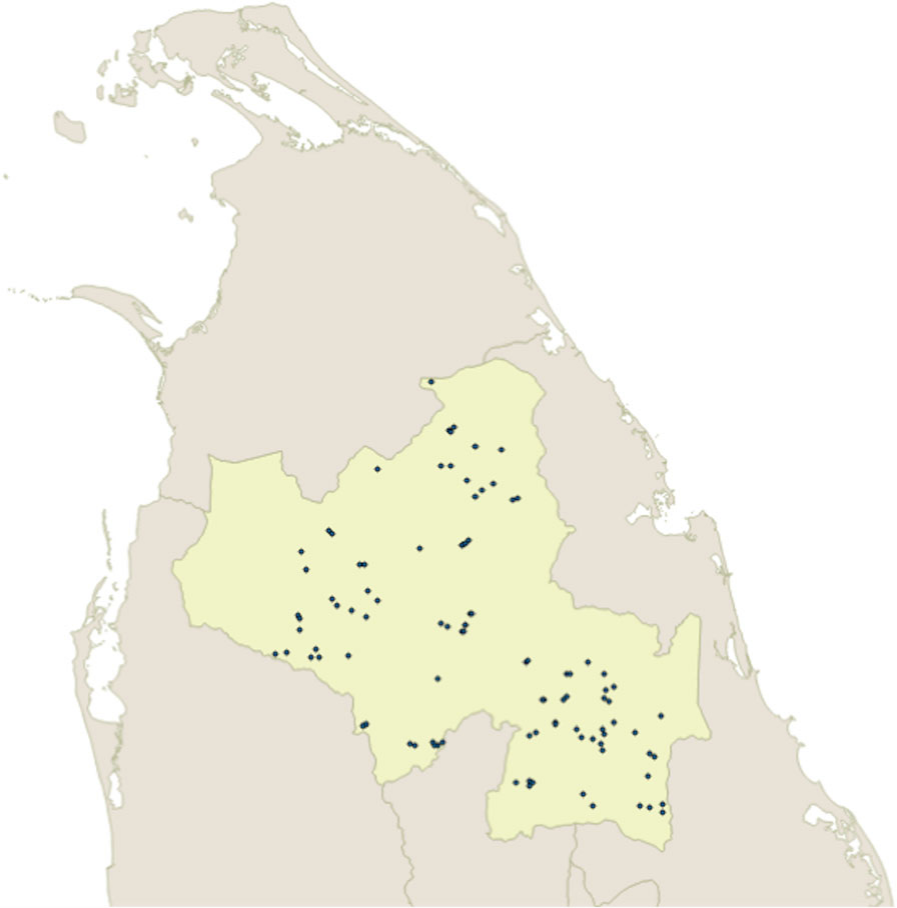
Distribution of 100 selected schools for nutritional assessment

We used a multi stage cluster sampling technique with probability proportionate to size. In the first stage, we calculated the number of schools to be selected from each educational zone using the number of students in each zone (Table 1). Within each educational zone, schools were listed according to the alphabetical order and a serial number was assigned. Within the each educational zone, a simple random sampling (SRS) technique was used to select the schools for the study. This was done using excel based random number generation to identify the serial numbers of schools to be selected. During the sampling process, we selected half the number of required schools using SRS method, which totaled for 50. The closest 50 schools for the selected 50 schools were selected using geographical locations to achieve the required number of 100 schools (Fig. 1). From the selected schools, we recruited a maximum number of 50 students using a simple random sampling technique using the school register as the sampling frame. We recruited all the students attending school if the total number of students is less than 50. Total sample size estimated was 4663 school children to represent 5% of the study population.

**Table 1:** Total number of eligible schools and the number of schools selected for the current study (based on student population proportions)

Educational zone	Total number of eligible schools	Number of schools selected
Anuradhapura	98	10
Thambuththegama	61	12
Galenbidunuwewa	90	12
Kekirawa	95	8
Kebithigollawa	120	16
Polonnaruwa	48	12
Hingurakgoda	91	18
Dimbulagala	98	12

### Study tools and measurements

A simple self-administered short questionnaire in Sinhalese and Tamil languages was used to assess the socio-demographic details. This questionnaire was filled by parents/guardians and therefore prepared simply so that parents with lowest education level can also fill it without any difficulty. All anthropometric measurements were done using WHO guide for anthropometric measurements by trained data collectors. A sample of 3 ml of venous blood was obtained from each child for detection of Haemoglobin level and for blood picture. Venepuncture was done by fully trained nurses. Analysis of heamoglobin level was done using fully automated analyzer in a laboratory with internal and external quality assurance procedures.

### Data collectors and data Collection

A team of 15 MBBS qualified medical graduates awaiting their internship appointments were trained on data collection, measurements and clinical assessment.

Questionnaires and consent forms were posted to schools 2 weeks prior to the date of data collection and the heads of the schools were contacted individually and were advised regarding distribution of questionnaires to parents and obtaining the consent. Questionnaires were accompanied by an information leaflet and parent consent form. Class teachers of selected children contacted the parents and guardians of children and explained the procedure and got the consent. The socio-demographic questionnaire was filled by the parents/guardians with the help of teachers.

### Data Analysis

We first calculated unadjusted, age and sex disaggregated descriptive statistics for weight, height, BMI, BMI for age (Z score), weight for age (z score), height for age (Z score) and mean heamoglobin concentration. Computation of individual Z scores was done using WHO Antho-Plus software in comparison to WHO child growth standards which were based on WHO Multicentre Growth Reference Study Group [23]. Since the indicator weight for age is recommended only up to the age of 9 years, we calculate that particular indicator only for the recommended age group. Since the study involved two-stages of sampling, we additionally used survey design adjusted estimates for selected age and sex specific estimates using linearized standard errors. For this, we used svy function of STATA 13 software with educational divisions as strata and schools as primary sampling units. In the second stage of sampling, adjustments were done for selection of students using the total number of classrooms with eligible students. Classification of anemia was done based on WHO recommendations for anemia in children classification; normal= > 12 g/dL, mild anemia 11.9-11.0 g/dL, moderate anemia 10.9-8.0 g/dL, severe anemia <8 g/dL. Hemoglobin and BMI z-score distribution maps were created using ArcGIS.

## Results

Of the 4633 students invited for the study, 4521 (97.6%) participated in the first stage; 2548 (56.5%) from Anuradhapura district and 1973 (43.5%) from Polonnaruwa. The age range of the study sample was 61 to 131 months. Male-female distribution was 2277 (50.4%) and 2227 (49.4%) (data missing in 17). Weight and height measurements were available for 4484 (99.2%) and 4435 (98.1%) children and the Hb data was available for 4412 children (97.6%).

Weight, height, BMI and Hb range in the study sample were 11–64.4 kg, 84.5–170 cm, 9.58–36.98Kgm^−2^ and 7.9 to 19.4 g/dl respectively. Distribution of height, weight, BMI and mean hemoglobin concentration by age shows a gradual increase as expected (Table 2).

**Table 2:** Distribution of weight, height, BMI, and mean hemoglobin concentration among school children in North Central Province, Sri Lanka

Age (months)	Weight (Kg)		Height (cm)		BMI		Hb (g/dL)	
	Mean	SD	Mean	SD	Mean	SD	Mean	SD
Males								
61–71	18.27	5.39	113.44	7.89	13.96	2.45	12.14	0.84
72–83	18.43	3.94	115.08	7.25	13.83	2.2	12.02	0.85
84–95	20.13	4.97	119.10	6.91	14.18	2.95	12.10	0.87
96–107	22.32	5.16	124.52	7.27	14.31	2.41	12.32	0.84
108–119	23.85	5.50	127.42	7.36	14.58	2.47	12.39	0.82
120–131	26.29	6.47	132.26	8.5	14.89	2.52	12.33	0.95
Females								
60–71	18.04	4.31	113.5	7.3	13.86	1.94	11.9	0.96
72–83	18.81	3.81	115.71	6.73	13.99	2.2	12.13	0.88
84–95	20.4	4.66	119.47	6.59	14.20	2.38	12.07	0.95
96–107	23.21	5.79	125.17	7.12	14.72	2.95	12.28	0.87
108–119	24.47	5.56	128.42	7.27	14.75	2.5	12.31	0.93
120–131	25.20	5.70	131.16	8.5	14.54	2.15	12.40	0.92

**Fig. 2:**
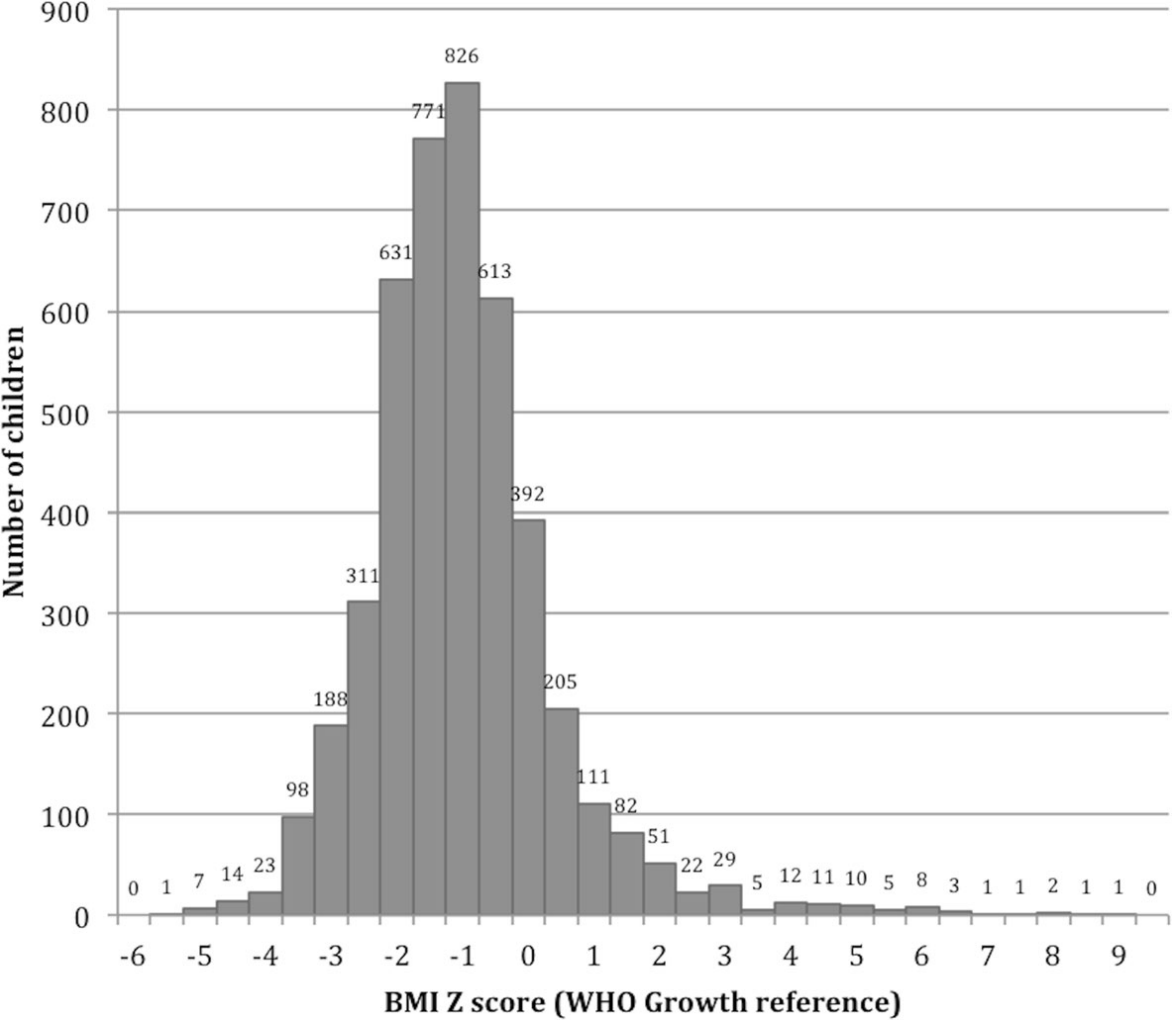
Distribution of BMI z scores among school children aging 5 to 10 years in North Central province, Sri Lanka

Distribution of BMI Z scores (Based on Z_ind_) in the study sample showed that the majority are scattered around −1 to −2 range (Fig. 2) and the graph was skewed to right. WHO AnthorPlus analysis also showed that the distribution of BMI Z- scores in this population is shifted towards the low side.

Prevalence of severe thinness, thinness, overweight and obesity in this study sample was 7.5% (*n* = 331), 21.2% (*n* = 942), 3.0% (*n* = 133) and 2.5% (*n* = 111) respectively. Age and sex disaggregated data on thinness showed a steady increase of both thinness (BMIZ < −2SD) and overweight/obesity (BMIZ > +1SD) from the age of 61 to 131 months (Table 3). More male children (*n* = 684, 30.5%) were having low BMI Z scores compared to female children (*n* = 584, 26.9%) and this difference was statistically significant (Chi-square 6.96, *p* < .031).

**Table 3:** Distribution of BMI Z values for age among school children aging 61 to 131 months in North Central province, Sri Lanka by age and sex

Age (Months)		BMI-for-age (%)											
	N	< −3SD	(95% CI)	<−2SD	(95% CI)	> +1SD	(95% CI)	> +2SD	(95% CI)	> +3SD	(95% CI)	Mean	SD
Male													
(61–71)	202	8.9	(4.7%, 13.1%)	29.2	(22.7%, 35.7%)	0.5	(0%, 1.7%)	0	(0%, 0.2%)	0	(0%, 0.2%)	−1.52	0.98
(72–83)	559	7.2	(4.9%, 9.4%)	26.3	(22.6%, 30%)	2.7	(1.3%, 4.1%)	1.4	(0.4%, 2.5%)	0.4	(0%, 0.9%)	−1.39	1.12
(84–95)	468	10.3	(7.4%, 13.1%)	31.8	(27.5%, 36.2%)	4.9	(2.8%, 7%)	2.6	(1%, 4.1%)	1.1	(0%, 2.1%)	−1.38	1.37
(96–107)	431	9.3	(6.4%, 12.1%)	30.6	(26.2%, 35.1%)	7.9	(5.2%, 10.5%)	4.6	(2.5%, 6.7%)	2.3	(0.8%, 3.9%)	−1.26	1.57
(108–119)	403	9.9	(6.9%, 13%)	33.5	(28.8%, 38.2%)	7.2	(4.5%, 9.8%)	2.7	(1%, 4.4%)	0.7	(0%, 1.7%)	−1.28	1.44
(120–131)	163	14.7	(9%, 20.5%)	39.9	(32.1%, 47.7%)	6.1	(2.1%, 10.1%)	1.8	(0%, 4.2%)	0	(0%, 0.3%)	−1.5	1.46
Female													
(61–71)	181	1.7	(0%, 3.8%)	22.1	(15.8%, 28.4%)	2.8	(0.1%, 5.4%)	1.1	(0%, 2.9%)	0	(0%, 0.3%)	−1.27	0.98
(72–83)	499	3.8	(2%, 5.6%)	23.6	(19.8%, 27.5%)	3.8	(2%, 5.6%)	1.6	(0.4%, 2.8%)	1	(0%, 2%)	−1.25	1.18
(84–95)	440	4.5	(2.5%, 6.6%)	26.4	(22.1%, 30.6%)	5.2	(3%, 7.4%)	2.3	(0.8%, 3.8%)	1.1	(0%, 2.2%)	−1.25	1.26
(96–107)	449	5.6	(3.3%, 7.8%)	26.1	(21.9%, 30.2%)	5.8	(3.5%, 8.1%)	1.8	(0.4%, 3.1%)	1.1	(0%, 2.2%)	−1.18	1.33
(108–119)	416	6.5	(4%, 9%)	26.7	(22.3%, 31.1%)	5.5	(3.2%, 7.8%)	1	(0%, 2%)	0.7	(0%, 1.7%)	−1.24	1.28
(120–131)	174	7.5	(3.3%, 11.7%)	32.2	(25%, 39.4%)	7.5	(3.3%, 11.7%)	1.7	(0%, 3.9%)	0	(0%, 0.3%)	−1.29	1.36
Total													
(61–71)	383	5.5	(3.1%, 7.9%)	25.8	(21.3%, 30.4%)	1.6	(0.2%, 2.9%)	0.5	(0%, 1.4%)	0	(0%, 0.1%)	−1.4	0.99
(72–83)	1058	5.6	(4.1%, 7%)	25.0	(22.4%, 27.7%)	3.2	(2.1%, 4.3%)	1.5	(0.7%, 2.3%)	0.7	(0.1%, 1.2%)	−1.33	1.15
(84–95)	908	7.5	(5.7%, 9.3%)	29.2	(26.2%, 32.2%)	5.1	(3.6%, 6.5%)	2.4	(1.4%, 3.5%)	1.1	(0.4%, 1.8%)	−1.32	1.32
(96–107)	880	7.4	(5.6%, 9.2%)	28.3	(25.3%, 31.3%)	6.8	(5.1%, 8.5%)	3.2	(2%, 4.4%)	1.7	(0.8%, 2.6%)	−1.22	1.45
(108–119)	819	8.2	(6.2%, 10.1%)	30.0	(26.8%, 33.2%)	6.3	(4.6%, 8.1%)	1.8	(0.9%, 2.8%)	0.7	(0.1%, 1.4%)	−1.26	1.36
(120–131)	337	11.0	(7.5%, 14.5%)	35.9	(30.6%, 41.2%)	6.8	(4%, 9.7%)	1.8	(0.2%, 3.3%)	0	(0%, 0.1%)	−1.39	1.41

Distribution of height for age showed an increasing trend of low height for age (stunting) with age. Total of 558 (12.6%) children were stunted and of them, 107 (2.4%) were having height for age Z-score less than −3 (severe stunting). No sex difference was observed for stunting in this study sample (Table 4).

**Table 4:** Distribution of height for age among school children aging 5 to 10 years in North Central province, Sri Lanka by age and sex

Age (Months)		Height-for-age (%)					
	N	< −3SD	(95% CI)	< −2SD	(95% CI)	Mean	SD
Male							
(61–71)	206	0.5	(0.0%, 1.7%)	10.7	(6.2%, 15.1%)	−0.66	1.11
(72–83)	565	1.6	(0.5%, 2.7%)	9.9	(7.4%, 12.5%)	−0.71	1.15
(84–95)	474	1.5	(0.3%, 2.7%)	12.4	(9.4%, 15.5%)	−0.87	1.05
(96–107)	432	1.6	(0.3%, 2.9%)	13.4	(10.1%, 16.8%)	−0.80	1.06
(108–119)	403	4.2	(2.1%, 6.3%)	13.2	(9.7%, 16.6%)	−0.93	1.08
(120–131)	164	7.9	(3.5%, 12.4%)	20.7	(14.2%, 27.2%)	−1.14	1.19
Female							
(61–71)	183	0.5	(0%, 1.9%)	7.7	(3.5%, 11.8%)	−0.47	1.28
(72–83)	500	1.4	(0.3%, 2.5%)	10.4	(7.6%, 13.2%)	−0.76	1.11
(84–95)	443	1.8	(0.5%, 3.2%)	9.3	(6.4%, 12.1%)	−0.74	1.02
(96–107)	449	1.1	(0.0%, 2.2%)	10.7	(7.7%, 13.7%)	−0.82	0.97
(108–119)	416	4.1	(2.1%, 6.1%)	16.6	(12.9%, 20.3%)	−1.06	1.11
(120–131)	175	7.4	(3.3%, 11.6%)	20.6	(14.3%, 26.8%)	−1.16	1.26
Total							
(61–71)	389	0.5	(0.0%, 1.4%)	9.3	(6.2%, 12.3%)	−0.57	1.2
(72–83)	1065	1.5	(0.7%, 2.3%)	10.1	(8.3%, 12.0%)	−0.73	1.13
(84–95)	917	1.6	(0.8%, 2.5%)	10.9	(8.8%, 13.0%)	−0.81	1.04
(96–107)	881	1.4	(0.5%, 2.2%)	12.0	(9.8%, 14.2%)	−0.81	1.02
(108–119)	819	4.2	(2.7%, 5.6%)	14.9	(12.4%, 17.4%)	−1.00	1.10
(120–131)	339	7.7	(4.7%, 10.6%)	20.6	(16.2%, 25.1%)	−1.15	1.22

Of the total sample, 1216 (27.1%) were having a weight for age Z- score less than −2 (Underweight). Prevalence of severe underweight was 7.0%. Distribution of underweight was similar in male and female children (Table 5).

**Table 5:** Distribution of Weight for age among school children aging 5 to 10 years in North Central province, Sri Lanka by age and sex

Age (Months)		Weight-for-age (%)					
	N	< −3SD	(95% CI)	< −2SD	(95% CI)	Mean	SD
Male							
(61–71)	204	5.4	(2%, 8.7%)	24.5	(18.4%, 30.7%)	−1.34	1.17
(72–83)	574	6.3	(4.2%, 8.3%)	28.0	(24.3%, 31.8%)	−1.32	1.21
(84–95)	478	10.3	(7.4%, 13.1%)	33.9	(29.5%, 38.2%)	−1.41	1.37
(96–107)	434	9.2	(6.4%, 12.1%)	28.6	(24.2%, 32.9%)	−1.30	1.38
(108–119)	404	0.5	(0%, 1.3%)	2.7	(1%, 4.4%)	−0.14	0.49
Female							
(61–71)	187	3.2	(0.4%, 6%)	23.5	(17.2%, 29.9%)	−1.17	1.24
(72–83)	512	6.8	(4.6%, 9.1%)	26.4	(22.5%, 30.3%)	−1.28	1.2
(84–95)	456	6.1	(3.8%, 8.5%)	25.7	(21.5%, 29.8%)	−1.28	1.2
(96–107)	453	6.4	(4%, 8.8%)	26.9	(22.7%, 31.1%)	−1.26	1.25
(108–119)	418	0.7	(0%, 1.6%)	3.1	(1.3%, 4.9%)	−0.16	0.54
Total							
(61–71)	391	4.3	(2.2%, 6.5%)	24.0	(19.7%, 28.4%)	−1.26	1.2
(72–83)	1086	6.5	(5%, 8.1%)	27.3	(24.6%, 30%)	−1.30	1.21
(84–95)	934	8.2	(6.4%, 10.1%)	29.9	(26.9%, 32.9%)	−1.34	1.29
(96–107)	887	7.8	(6%, 9.6%)	27.7	(24.7%, 30.7%)	−1.28	1.32
(108–119)	822	0.6	(0%, 1.2%)	2.9	(1.7%, 4.1%)	−0.15	0.51

**Fig. 3:**
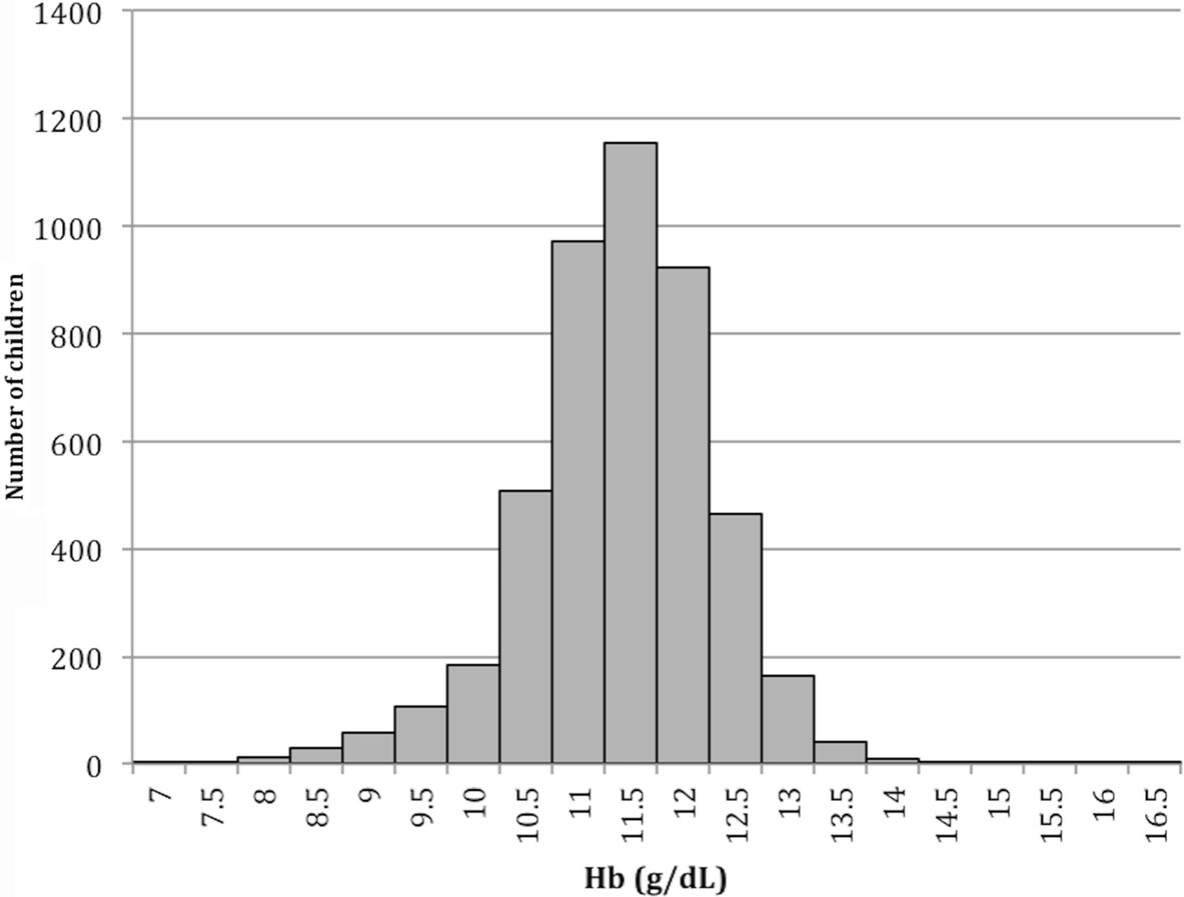
Distribution of mean hemoglobin concentration among school children aging 5 to 10 years in North Central province, Sri Lanka

Distribution of mean Hb values was skewed toward to left (Fig. 3). Mean Hb concentration was 12.19 g/dL (SD .89 g/dL) with no significant sex difference.

Prevalence of anemia was 17.1% (*n* = 756) in this study sample. Only a single child had severe anemia with a Hb of 7.9 mg/dl. Prevalence of mild and moderate anemia was 9.4% (*n* = 416) and 7.6%(*n* = 336). Among both male and female children, a clear declining of anemic status was noted from 23.6% at the age of 61–72 months to 12.6% at 120–131 months (Table 6).

**Table 6:** Distribution of anemia among school children aging 5 to 10 years in North Central province, Sri Lanka by age and sex

	Age (months)	Total number studied	Normal ^a^		Mild anemia		Moderate anemia	
			n	%	n	%	n	%
Male	61–71	204	152	74.5	29	14.2	22	10.8
	72–83	563	461	81.9	56	9.9	46	8.2
	84–95	470	368	78.3	54	11.5	48	10.2
	96–107	439	377	85.9	38	8.7	24	5.5
	108–119	406	350	86.2	23	5.7	33	8.1
	120–131	146	128	87.7	6	4.1	12	8.2
	*Total*	2228	*1836*	*82.4*	*206*	*9.2*	*185*	*8.3*
Female	61–71	182	143	78.6	20	11.0	19	10.4
	72–83	505	391	77.4	67	13.3	47	9.3
	84–95	455	371	81.5	41	9.0	43	9.5
	96–107	444	388	87.4	40	9.0	16	3.6
	108–119	402	360	89.6	28	7.0	14	3.5
	120–131	180	157	87.2	12	6.7	11	6.1
	*Total*	2168	*1810*	*83.5*	*208*	*9.6*	*150*	*6.9*

^a^ Classification is based on WHO recommendations for childhood anemia. Normal = <12 g/dL, mild anemia 11.9-10.0 g/dL, moderate anemia 10.9-80 g/dL, severe anemia <8 g/dL

**Fig. 4:**
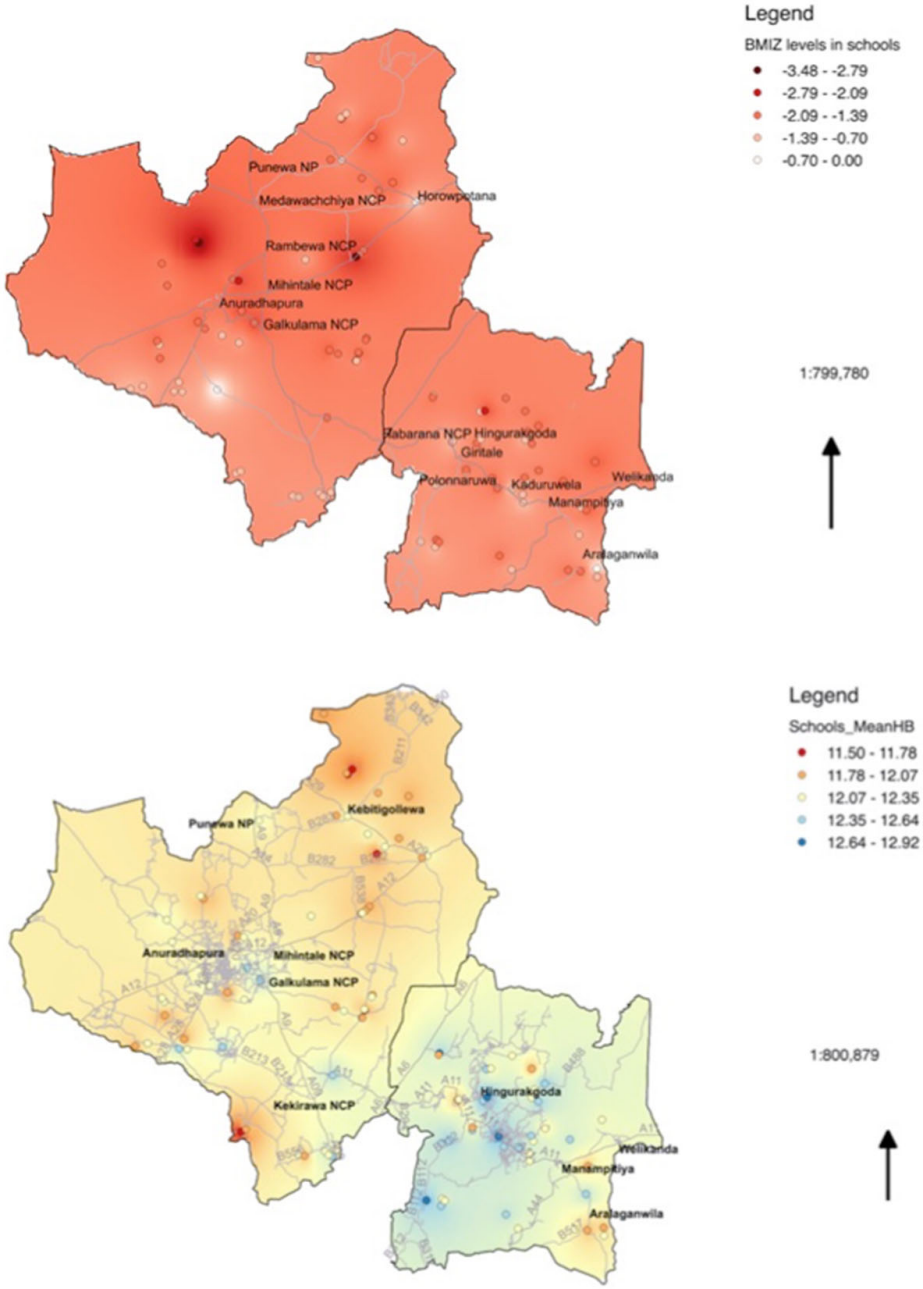
Geographical distribution of mean haemoglobin values and mean BMI Z scores among 100 selected schools in North Central province, Sri Lanka

Geographical distribution of thinness and hemoglobin concentration showed that these nutritional problems are spatially clustered (Fig. 4). Both anemia and thinness were more prevalent in schools away from the main cities. Anuradhapura district had a high prevalence of anemia (20.1% compared to 13.1% in Polonnaruwa district) and also thinness (34.8% compared to 31.7%).

We estimated the survey design adjusted prevalence of thinness, wasting, stunting and anemia (Table 7). Adjusted mean estimates for hemoglobin and BMI Z scores in this study population were 12.20 (95% CI 12.16-12.24) g/dL and −1.421 (95% CI −1.578 -1.262 respectively.

**Table 7:** Survey design adjusted estimates for thinness, stunting, underweight and anemia among school children aging 5 to 10 years in North Central province, Sri Lanka

	Adjusted Prevalence	Linearized Std. Err.	95% Conf. Intervals	
Thinness				
Severe thinness	8.60	0.94	6.91	10.66
Thinness	20.91	0.74	19.48	22.40
Normal BMI	65.11	1.31	62.46	67.67
Overweight	2.95	0.26	2.47	3.52
Obese	2.44	0.92	1.15	5.10
Stunting				
No stunting	56.08	1.23	53.62	58.51
Stunting	30.32	0.83	28.69	32.01
Severe stunting	13.60	0.93	11.86	15.55
Underweight				
No underweight	74.07	0.96	72.11	75.93
Underweight	25.93	0.96	24.07	27.89
Anemia				
Non-anemic	83.09	0.69	81.68	84.41
Mild	9.33	0.49	8.39	10.36
Moderate	7.56	0.46	6.69	8.53
Severe	0.02	0.02	0.00	0.14

## Discussion

Recent studies from high and middle-income countries such as Sweden (16.5–25.7%) [24], India (11.7%) [25], China (14.3–20%) [26] and Nigeria (15.5%) [27] clearly shows an alarmingly high percentage of obesity among pre-adolescent age group. However, more children in the same age group are reported as having low BMI for age in studies from low income countries such as Sudan [28], Tanzania and Burma [29]. However, prevalence of anemia reported in this study from rural Sri Lanka was less than many other reports from low and middle income. The absence of sex disparity in nutritional status was showing the universal coverage of services and minimal gender based discrimination for female children in this setting.

The target age group in the present study was not extensively studied in the local context during recent years. In a study done in Colombo district in 2004 among children aging 8–12 years reported a slightly higher prevalence of obesity (boys 4.3% and girls 3.1%), lower prevalence of thinness (24.7% in boys and 23.1% in girls) and lower prevalence of stunting (5.1% of boys and 5.2% of girls) showing an obvious disparity between urban and rural school children even with 12 years time gap [30]. If an increase of nutritional status is expected over these 12 years, higher disparities could have been observed among rural North Central Schools and urban Colombo schools.

Increasing prevalence of macro-nutrition problems with age, reducing prevalence of anemia and increase prevalence of obesity was observed in all demographic health surveys from 1993 to 2006 and the most recent survey by MRI 2009 [8, 14, 15, 31]. Male predominance of nutrition problems was also observed in previous studies [8]. Underlying reasons for increasing energy deficiency (both acute and chronic) among children is yet to be investigated properly. The most recent estimates of low birth weight for Sri Lanka is 15.8% showing a lesser degree problem of energy malnutrition at birth. While the school health program specifically looks into nutritional problems as a priority area, high and increasing level of thinness and stunting with age needs urgent attention and proper investigations to develop effective public health interventions.

Geographical disparities in nutrition within countries are well-known. However, the distribution of nutritional problems observed in this study is different from all DHS surveys and recent studies. Studies done in 2006, 2009 and 2012 shows a high prevalence of under five malnutrition and anemia in Polonnaruwa district [14, 15, 31]. In our study, all indicators were better in Polonnaruwa district. The present study is the largest study done in this area and involving 100 schools. The only probable explanation is that the previous studies may have included large schools located within the urban areas and according to the educational department classification of schools, only a single school in Polonnaruwa is excluded from this study, while a large number of urban schools from Anuradhapura were excluded. However, our data on rural schools are from a better sampling frame and are based on much larger sample compared to all the other published studies from this area.

Interpretation of the findings of this study should be done with careful evaluation of the methodology. First, the study sample was school based, but not community based. Even though the primary enrolment 99.8% and almost all of they remain at school at grade 5, a small sample, who are socio economically deprived may have been missed from this study, slightly underestimating the nutritional problems. However, the selection of difficult to very difficult schools may have systematically excluded larger, urban schools in the province and the estimated anemia and BMI Z scores may be an overestimation and or the results are valid only for rural schools. In addition, clustering of health condition may also have an effect due to the design we used, which is a known error in these type of studies and unavoidable.

## Conclusion

More than one-third of children aging 5–10 years in rural NCP are having low BMI and one-sixth have anemia. The increasing prevalence of thinness and obesity with age needs urgent attention at national level. Further studies are recommended to explore the underlying factors of nutritional problems in pre-adolescent age, in order to formulate evidence based strategies to combat nutritional problems among children in pre-adolescent age in Sri Lanka.

## Abbreviations

BMI: Body mass index
CI: Confidence intervals
DHS: Demographic and health survey
Hb: Haemoglobin
MRI: Medical Research Institute
NCP: North Central Province
SE: Standard error
WHO: World Health Organization
